# Lactate promotes *Salmonella* intracellular replication and systemic infection via driving macrophage M2 polarization

**DOI:** 10.1128/spectrum.02253-23

**Published:** 2023-10-05

**Authors:** Xinyue Wang, Bin Yang, Shuangshuang Ma, Xiaolin Yan, Shuai Ma, Hongmin Sun, Yuyang Sun, Lingyan Jiang

**Affiliations:** 1 The Key Laboratory of Molecular Microbiology and Technology, Ministry of Education, TEDA Institute of Biological Sciences and Biotechnology, Nankai University, Tianjin, China; 2 Department of Biopharmaceuticals, Tianjin Key Laboratory on Technologies Enabling Development of Clinical Therapeutics and Diagnostics, School of Pharmacy, Tianjin Medical University, Tianjin, China; South China Sea Institute of Oceanology, Chinese Academy of Sciences, Guangzhou, Guangdong, China

**Keywords:** *Salmonella*, macrophages, lactate, infection, macrophage polarization

## Abstract

*Salmonella* is one of the most important enteric pathogens worldwide, which is able to cause lethal systemic infection via survival and replication in host macrophages. Lactate, a byproduct of anaerobic or aerobic glycolysis, can induce macrophage M2 polarization, but the relationship between lactate-mediated macrophage M2 polarization and bacterial infection is poorly understood. Here, we evaluated the role of lactate and lactate-mediated macrophage M2 polarization in the pathogenicity of *Salmonella*. We found that lactate levels were significantly increased in *Salmonella*-infected macrophages, and the increased lactate was derived from the host. Macrophage and mouse infection assays showed that the addition of lactate enhanced *Salmonella* replication within macrophages and the colonization of mouse systemic loci, while pharmacological or genetic inhibition of host lactate production impaired *Salmonella* intracellular replication and its virulence in mice. Further analysis revealed that lactate promotes M2 polarization of *Salmonella*-infected macrophages, and the induction of macrophage M2 polarization by lactate is responsible for lactate-mediated *Salmonella* growth promotion. Moreover, we showed that macrophage-derived lactate induces the *Salmonella* pathogenicity island (SPI)-2 type III secretion system, leading to increased translocation of the SPI-2 effector SteE, which is responsible for driving M2 polarization. Overall, these findings suggest that lactate promotes *Salmonella* intracellular replication and systemic infection via driving macrophage M2 polarization and highlight the complex interactions between *Salmonella* and macrophages.

**IMPORTANCE:**

The important enteropathogen *Salmonella* can cause lethal systemic infection *via* survival and replication in host macrophages. Lactate represents an abundant intracellular metabolite during bacterial infection, which can also induce macrophage M2 polarization. In this study, we found that macrophage-derived lactate promotes the intracellular replication and systemic infection of *Salmonella*. During *Salmonella* infection, lactate *via* the *Salmonella* type III secretion system effector SteE promotes macrophage M2 polarization, and the induction of macrophage M2 polarization by lactate is responsible for lactate-mediated *Salmonella* growth promotion. This study highlights the complex interactions between *Salmonella* and macrophages and provides an additional perspective on host-pathogen crosstalk at the metabolic interface.

## INTRODUCTION

*Salmonella* is a major enteric pathogen that infects humans and animals, causing self-limiting enteritis or severe fatal systemic diseases (1, 2). *Salmonella* infection remains a serious health problem worldwide, with more than 100 million cases annually, resulting in about 350,000 deaths (3, 4). Pathogenesis of *Salmonella* infections involves two critical steps: invasion into intestinal epithelial cells and replication within host myeloid cells, notably macrophages (5 – 8). Replication within host macrophages is essential for systemic infection (9 – 11). After being engulfed by macrophages, *Salmonella* resides within a specialized membrane-bound compartment termed the *Salmonella*-containing vacuole (SCV), which facilitates *Salmonella* evasion of macrophage antibacterial responses and the efficient acquisition of nutrients (12 – 14). The biogenesis and maturation of SCV mainly rely on the function of a type III secretion system (T3SS) encoded by *Salmonella* pathogenicity island-2 (SPI-2) (5, 15).

Macrophages are innate immune cells constituting the first line of defense against an infection (16). The physiological state of macrophages is plastic and regulated by microenvironmental stimuli (17). Depending on the activation state, the macrophages are divided into classically activated M1 macrophages and alternatively activated M2 macrophages, which generally exert host defense and tissue repair functions, respectively (18). After stimulation by pathogen-associated molecular patterns [such as lipopolysaccharide (LPS)] and/or by interferons (such as IFN-γ), macrophages are polarized to M1 phenotype, which produce high levels of nitric oxide and proinflammatory cytokines against microbial infection. In contrast, stimulation of macrophages using cytokines such as interleukin (IL)-4 and IL-13 promotes anti-inflammatory response, and macrophages are polarized to the M2 phenotype to promote tissue repair (19, 20). *Salmonella* mainly resides and grows in M2 macrophages rather than in M1 macrophages (21 – 23). Moreover, *Salmonella* can actively promote macrophage M2 polarization by activating signal transducers and activators of transcription 3 (STAT3), via the SPI-2 T3SS effector SteE (24, 25).

Lactate or lactic acid, a byproduct of anaerobic or aerobic glycolysis (the Warburg effect), is produced from the conversion of pyruvate by lactate dehydrogenase A (LDHA) (26, 27). During bacterial infection, lactate production is significantly increased in infected macrophages, due to the reprogramming of cellular glucose metabolism from oxidative phosphorylation (OXPHOS) to aerobic glycolysis (28). Several intracellular bacterial pathogens, such as *Mycobacterium tuberculosis* and *Brucella abortus,* can exploit macrophage-derived lactate as a nutrient to grow and survive in the intracellular niche (29, 30). Although lactate levels are increased in *Salmonella*-infected macrophages, lactate is not exploited by intracellular *Salmonella* as a nutrient; instead, *Salmonella* senses host-derived lactate as a host cue to induce its SPI-2 T3SS expression (31). However, there is still no direct evidence to support that lactate promotes *Salmonella* replication in macrophages.

Emerging evidence suggests that lactate can promote macrophage polarization to M2 phenotype, via mechanisms such as activation of hypoxia-inducible factor-1α and ERK/STAT3 signaling pathway (32, 33). Given that *Salmonella* mainly grows in M2 macrophages, it is unclear whether lactate can promote *Salmonella* replication in macrophages by promoting macrophage M2 polarization. Both lactate and *Salmonella* can promote macrophage M2 polarization, but the relationship between lactate- and *Salmonella*-mediated M2 polarization is unknown.

In this study, we aimed to investigate the effects of lactate and lactate-mediated macrophage M2 polarization on *Salmonella* intracellular replication and pathogenicity. Through *in vitro* and *in vivo* infection assays along with seahorse analysis, flow cytometry, and many other molecular techniques, we report that macrophage-derived lactate promotes *Salmonella* replication within macrophages and colonization of mouse systemic loci. During *Salmonella* infection, lactate via the *Salmonella* SPI-2 T3SS effector SteE promotes macrophage M2 polarization, and the induction of macrophage M2 polarization by lactate is responsible for lactate-mediated *Salmonella* growth promotion. Our findings illustrate the important role of host-derived lactate in *Salmonella* pathogenicity and highlight the complex interactions between *Salmonella* and macrophages.

## RESULTS

### Lactate promotes *Salmonella* replication within macrophages

**Fig 1:**
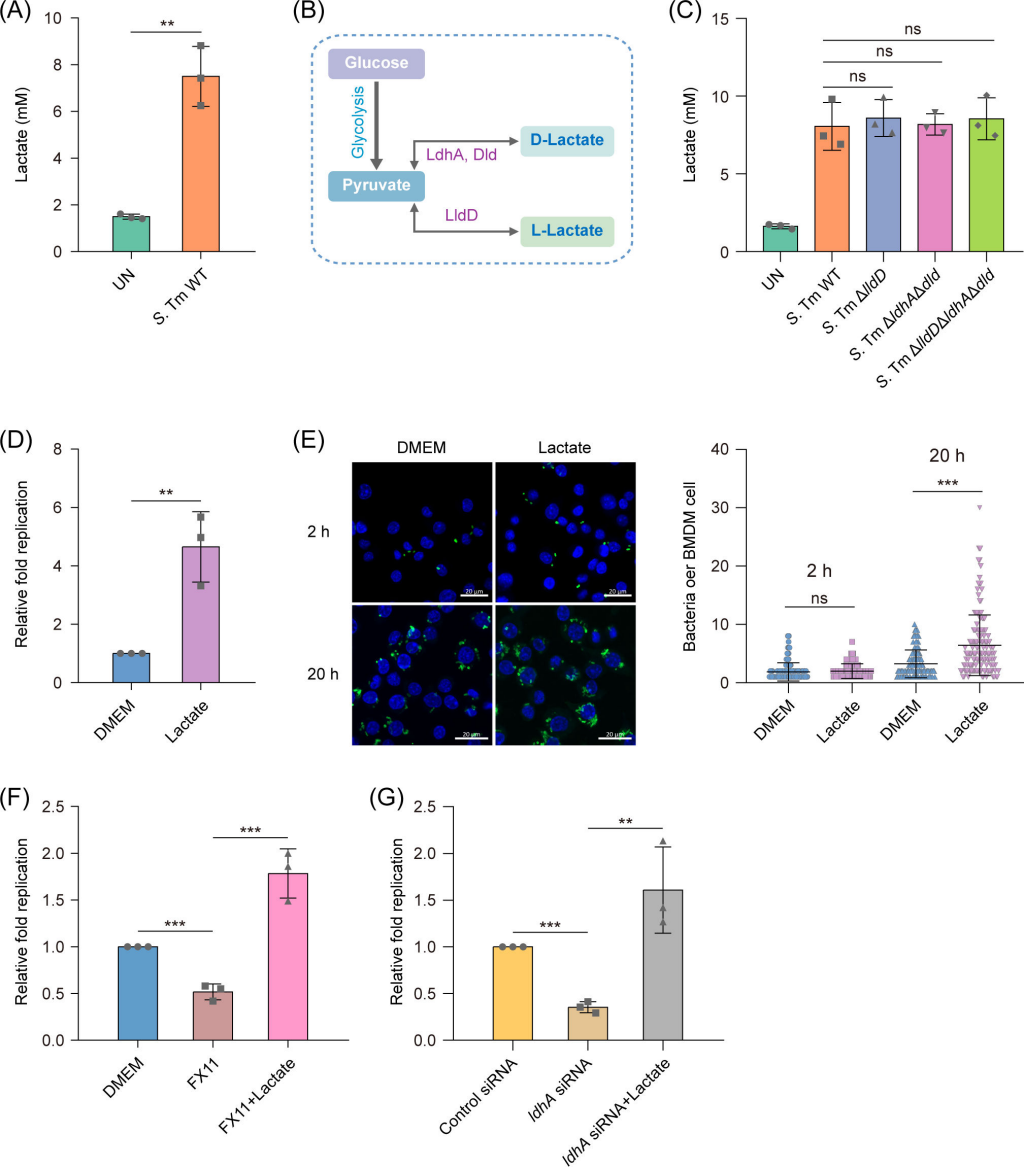
Lactate promotes *Salmonella* replication within macrophages. (**A**) Lactate production by uninfected BMDMs (UN) or those infected with *Salmonella* WT (*Salmonella* Typhimurium ATCC 14028s) for 20 h. (**B**) Enzymes responsible for D- and L-lactate production in *Salmonella*. (**C**) Lactate production by uninfected BMDMs (UN) or those infected with *Salmonella* WT, Δ*lldD*, Δ*ldhA*Δ*dld*, and Δ*lldD*Δ*ldhA*Δ*dld* for 20 h. (**D**) Replication of *Salmonella* WT in BMDMs, in the presence or absence of 3 mM lactate. (**E**) Number of intracellular bacteria per BMDM, in the presence or absence of 3 mM lactate. Infected cells were fixed at 2 and 20 h post-infection and prepared for immunofluorescence staining. The number of intracellular bacteria per infected cell was counted in random fields, *n* = 150 cells per group pooled from three independent experiments. Representative immunofluorescence images were shown in the left panel. Green, *Salmonella*; blue, nuclei. Scale bars, 20 µm. (**F**) Replication of *Salmonella* WT in BMDMs, in the presence or absence of 23.3 µM FX11 and 23.3 µM FX11+ 3 mM lactate. (**G**) Replication of *Salmonella* WT in *ldhA* siRNA-treated or control siRNA-treated RAW264.7 cells, in the presence or absence of 3 mM lactate. Data are presented as mean ± SD of three independent experiments (A, C–F). *P* values were determined using two-tailed unpaired Student’s *t*-test (**A, D, and E**) or one-way analysis of variance (ANOVA) (**C, F, and G**). ^**^ *P* < 0.01, ^***^ *P* < 0.001; ns, not significant.

Previous studies have reported that the lactate levels of macrophages were increased in response to *Salmonella* infection (31), which was also confirmed here. At 20 h post-infection with wild-type *Salmonella* (*Salmonella enterica* serovar Typhimurium ATCC 14028s, WT), the lactate levels of mouse primary bone-marrow-derived macrophages (BMDMs) increased 4.8-fold relative to those of uninfected BMDMs (Fig. 1A). Lactate levels in WT-infected BMDMs are comparable to that of the BMDMs infected with three mutant strains, Δ*lldD* (cannot produce L-lactate), Δ*ldhA*Δ*dld* (cannot produce D-lactate), and Δ*lldD*Δ*ldhA*Δ*dld* (cannot produce D- and L-lactate) (Fig. 1B and C), indicating that the increased lactate in infected BMDMs is derived from the macrophages.

We analyzed the effect of lactate on *Salmonella* replication in macrophages. The replication of *Salmonella* in BMDMs was significantly (*P* < 0.01) increased with the addition of 3 mM lactate (Fig. 1D). Immunofluorescence analysis showed that the addition of lactate did not influence the number of bacteria in each infected BMDM at initial infection stage (2 h) but increased the number of bacteria in each infected BMDM at 20 h post-infection (Fig. 1E). When we inhibited lactate production in BMDMs by adding the competitive inhibitor FX11 (23.3 µM) of LDHA, *Salmonella* replication decreased 2.2-fold, and lactate addition relieved the bacterial replication defect mediated by FX11 (Fig. 1F). siRNA knockdown of LDHA also decreased *Salmonella* replication in mouse RAW264.7 macrophage cell line, and lactate addition relieved the bacterial replication defect mediated by *ldhA* siRNA (Fig. 1G). These results collectively indicate that macrophage-derived lactate promotes *Salmonella* replication within macrophages.

### Lactate promotes *Salmonella* colonization of systemic loci in mice

**Fig 2:**
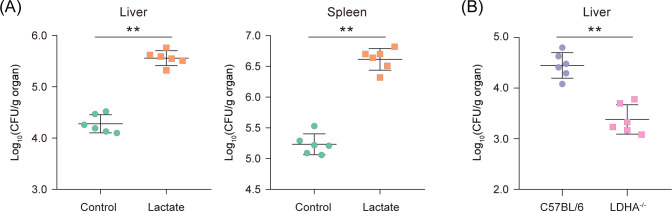
Lactate promotes *Salmonella* colonization of systemic loci in mice. (**A**) Bacterial counts recovered from the liver and spleen of C57BL/6 mice intraperitoneally (i.p.) infected with *Salmonella* WT. Mice were either i.p injected with ~10^4^ CFU bacteria plus lactate (0.6 mg lactate/ g mice) or bacteria only (control). Liver and spleen were collected on day 3 post-infection to quantify bacterial burden; *n* = 6 mice per group. (**B**) Bacterial counts recovered from the liver of wild-type or liver-specific LDHA knockout (LDHA^−/−^) C57BL/6 mice i.p. infected with *Salmonella* WT. Mice were i.p injected with ~10^4^ CFU bacteria. The liver was collected on day 3 post-infection to quantify bacterial burden; *n* = 6 mice per group. Data are combined from two independent experiments (**A, B**). *P* values were determined using Mann-Whitney U test (**A, B**). ^**^ *P* < 0.01.

To assess whether lactate contributes to *Salmonella* infection *in vivo*, we injected *Salmonella*-infected mice with lactate (0.6 mg lactate/g mice) or phosphate-buffered saline (PBS) (control). The livers and spleens of infected mice were collected on day 3 post-infection to quantify the bacterial burden. The results showed that lactate injection significantly (*P* < 0.01) increased the bacterial burden in the mouse liver and spleen (Fig. 2A). Moreover, the bacterial burden was significantly (*P* < 0.01) less in the livers of liver-specific LDHA knockout mice (LDHA^−/−^) compared to that in C57BL/6 wild-type mice (Fig. 2B). In contrast, the bacterial burden in the spleens of LDHA^−/−^ mice is comparable to that of wild-type mice (Fig. S1), suggesting that the decreased bacterial colonization in LDHA^−/−^ mice is a liver-specific phenotype, as a result of *ldhA* knock out in the liver. These results indicate that lactate promotes *Salmonella* colonization of systemic loci in mice.

### Lactate promotes M2 polarization of *Salmonella*-infected macrophages

**Fig 3:**
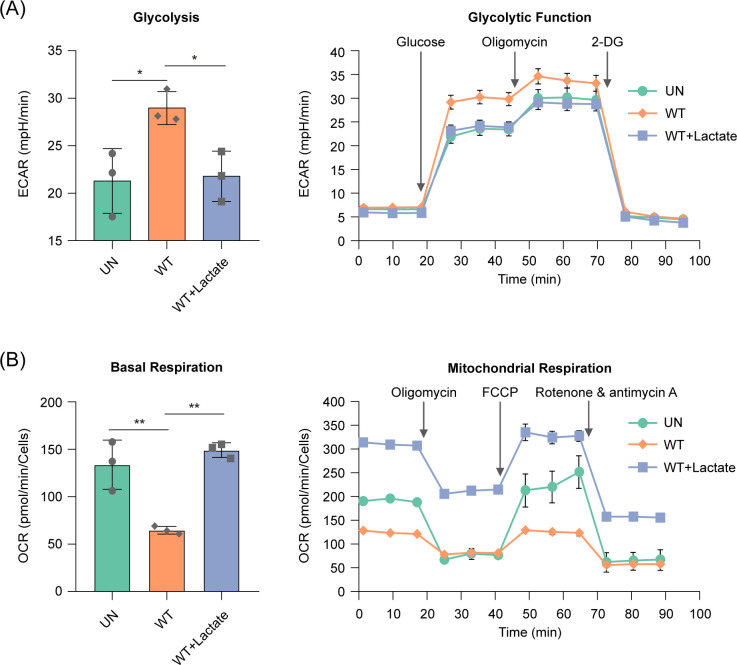
Lactate inhibits glycolysis and promotes oxidative phosphorylation (OXPHOS) of *Salmonella*-infected macrophages. Extracellular acidification rate (ECAR) (**A**) and oxygen consumption rate (OCR) (**B**) of uninfected BMDMs (UN) or *Salmonella* WT-infected BMDMs at 20 h post-infection (hpi), in the presence or absence of 3 mM lactate (left). Real-time changes in the ECAR and OCR were shown in the right panel of (**A**) and (**B**), respectively. 2-DG, 2-deoxy-D-glucose; FCCP, carbonyl cyanide p-(tri-fluromethoxy) phenyl-hydrazone. Data are presented as mean ± SD of three independent experiments (**A, B**). *P* values were determined using one-way ANOVA (**A, B**). ^*^ *P* < 0.05, ^**^ *P* < 0.01.

As lactate can promote macrophage M2 polarization, we then assessed whether lactate promotes M2 polarization of *Salmonella*-infected macrophages. Energy metabolism is closely related to the polarized phenotype of macrophages, with M1 macrophages exhibiting enhanced glycolysis and reduced OXPHOS, whereas M2 macrophages display enhanced OXPHOS and reduced glycolytic metabolism (34). Seahorse analysis showed that extracellular acidification rate (ECAR), an indicator of glycolytic flux, was significantly (*P* < 0.05) increased in *Salmonella* WT-infected BMDMs compared to the uninfected BMDMs (Fig. 3A), while basal oxygen consumption rate (OCR), an indicator of mitochondrial respiration, was significantly (*P* < 0.01) decreased in *Salmonella* WT-infected BMDMs compared to that of the uninfected BMDMs (Fig. 3B), indicating a metabolic reprogramming of macrophages from OXPHOS to glycolysis upon *Salmonella* infection, which are in agreement with previous results (31). The addition of 3 mM lactate decreased the ECAR and increased the OCR of *Salmonella* WT-infected BMDMs (Fig. 3A and B). These results suggest that lactate inhibits glycolysis and promotes OXPHOS in *Salmonella*-infected macrophages. The repression of glycolysis and promotion of OXPHOS by lactate indicate an M2 bias in *Salmonella*-infected macrophages in the presence of lactate.

**Fig 4:**
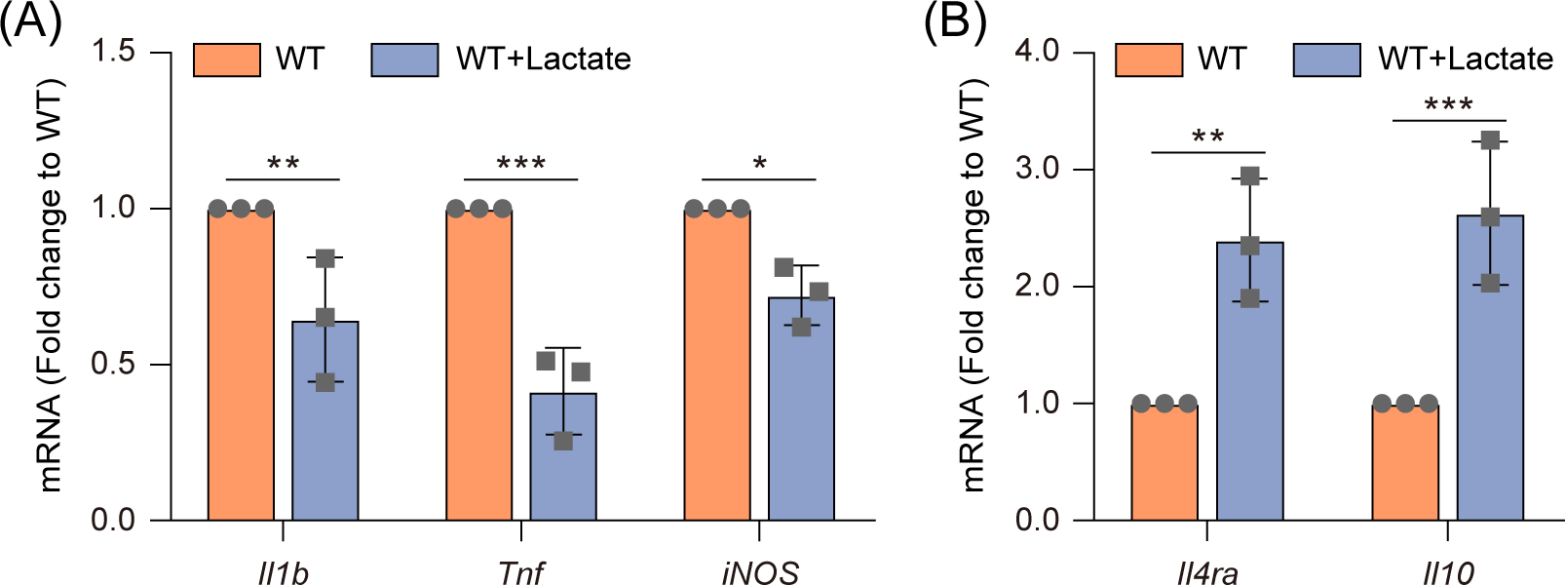
Lactate inhibits M1 polarization gene expression and promotes M2 polarization gene expression of *Salmonella*-infected macrophages. Quantitative real-time PCR (qRT-PCR) analysis of the mRNA levels of M1 marker genes (*Il1b*, *Tnf*, and *iNOS*) (**A**) and M2 marker genes (*Il4ra* and *Il10*) (**B**) in *Salmonella* WT-infected RAW264.7 cells at 20 hpi, in the presence or absence of 3 mM lactate. Data are presented as mean ± SD of three independent experiments (**A, B**). *P* values were determined using two-way ANOVA (**A, B**). ^*^ *P* < 0.05, ^**^ *P* < 0.01, ^***^ *P* < 0.001.

Moreover, we analyzed the expression levels of three M1 macrophage marker genes (*Il1b*, *Tnf*, and *iNOS*) and two M2 macrophage marker genes (*Il4ra* and *Il10*) in *Salmonella* WT-infected RAW264.7 macrophages, in the presence or absence of 3 mM lactate. The results showed that upon the addition of lactate, the expression of M1 marker genes was significantly decreased, while the expression of M2 marker genes was significantly (*P* < 0.05) increased in *Salmonella* WT-infected RAW264.7 macrophages (Fig. 4A and B), indicating that lactate inhibits M1 polarization gene expression and promotes M2 polarization gene expression of *Salmonella*-infected macrophages.

Collectively, these results indicate that lactate promotes M2 polarization of *Salmonella*-infected macrophages.

### Lactate promotes *Salmonella* intracellular replication via driving macrophage M2 polarization

**Fig 5:**
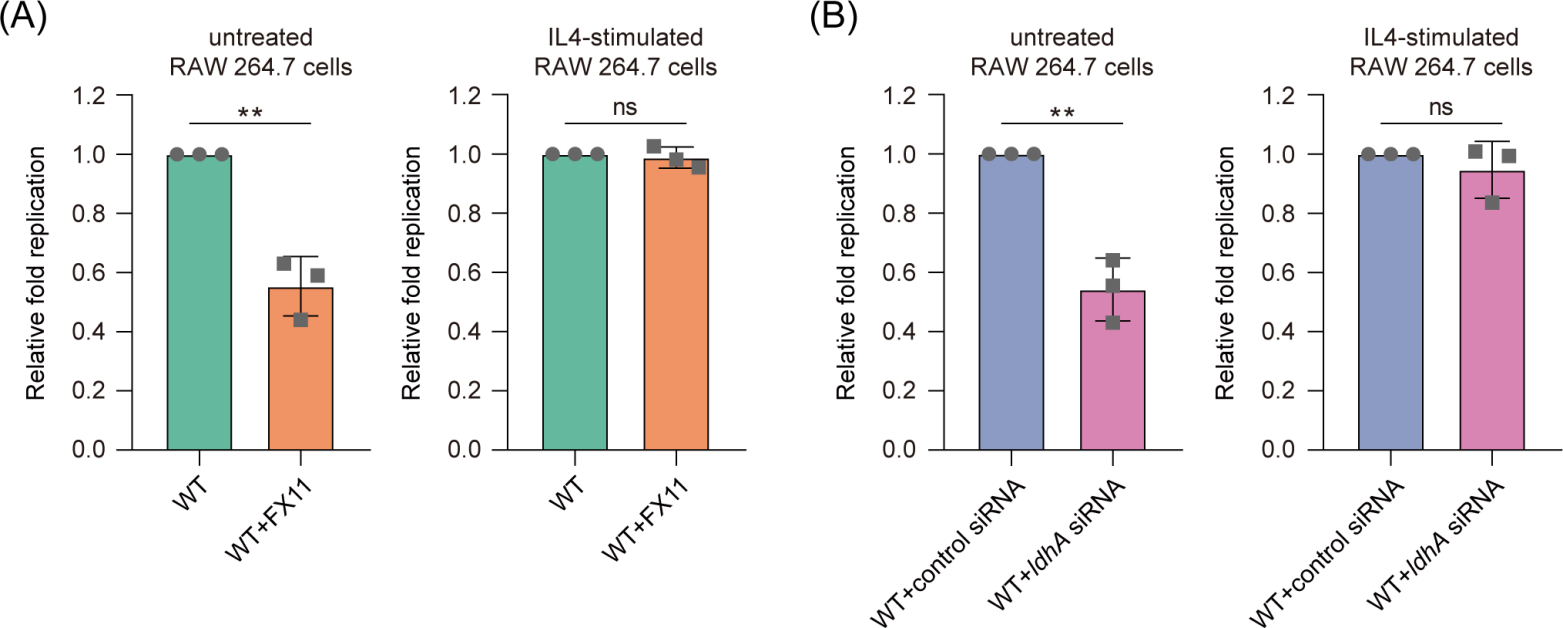
Lactate promotes *Salmonella* intracellular replication via driving macrophage M2 polarization. (**A**) Replication of *Salmonella* WT in untreated (left panel) and IL4-stimulated (right panel) RAW264.7 cells, in the presence or absence of 23.3 µM FX11. RAW264.7 cells were left untreated or cultured for 24 h with IL4 (10 ng/mL) to induce M2 polarization, and then, the cells were infected with *Salmonella* WT. (**B**) Replication of *Salmonella* WT in *ldhA* siRNA-treated or control siRNA-treated RAW264.7 cells. RAW264.7 cells were left untreated (left panel) or cultured for 24 h with IL4 to induce M2 polarization (right panel). Data are presented as mean ± SD of three independent experiments (**A, B**). *P* values were determined using two-tailed unpaired Student’s *t*-test (**A, B**). ^**^ *P* < 0.01. ns, not significant.

Next, we assessed the relationship between lactate-mediated macrophage M2 polarization and *Salmonella* growth promotion. We treated RAW264.7 macrophages with IL4 to induce M2 polarization, and then, the IL4-stimulated macrophages were infected with *Salmonella* WT to test bacterial replication ability in the presence or absence of the LDHA inhibitor FX11, which inhibits macrophage lactate production. Addition of FX11 inhibited *Salmonella* replication in RAW264.7 cells (Fig. 5A, left panel). If lactate promotes *Salmonella* intracellular replication via driving macrophage M2 polarization, the decreased replication ability of *Salmonella* due to decreased intracellular lactate levels could be offset by the pre-induction of macrophages into the M2 phenotype. As expected, the addition of FX11 did not influence the replication ability of *Salmonella* in IL4-stimulated RAW264.7 cells (Fig. 5A, right panel). Moreover, siRNA knockdown of LDHA decreased *Salmonella* replication in RAW264.7 cells (Fig. 5B, left panel), but upon pre-stimulation with IL4, the replication ability of *Salmonella* WT in *ldhA* siRNA-treated RAW264.7 cells was comparable to that of the control siRNA-treated cells (Fig. 5B, right panel), indicating that the pre-stimulation of macrophages to the M2 phenotype abolished the bacterial replication defect mediated by *ldhA* siRNA. These results suggest that macrophage-derived lactate promotes *Salmonella* intracellular replication via driving macrophage M2 polarization.

### Lactate promotes macrophage M2 polarization via the SPI-2 T3SS effector SteE

**Fig 6:**
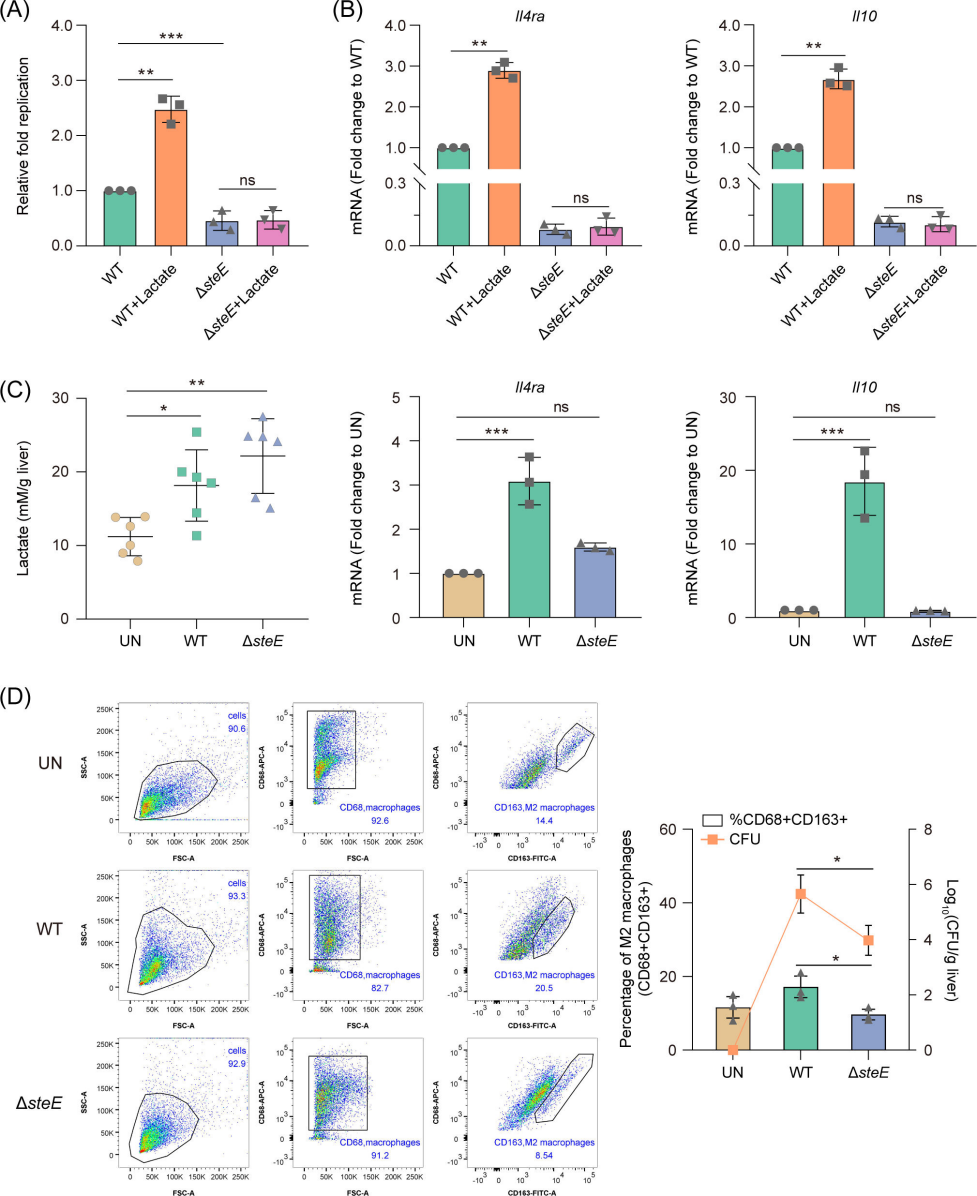
Lactate promotes macrophage M2 polarization via the SPI-2 T3SS effector SteE. (**A**) Replication of *Salmonella* WT or Δ*steE* in RAW264.7 cells, in the presence or absence of 3 mM lactate. (**B**) qRT-PCR analysis of the mRNA levels of M2 marker genes (*Il4ra* and *Il10*) in *Salmonella* WT-infected or Δ*steE*-infected RAW264.7 cells at 20 hpi, in the presence or absence of 3 mM lactate. (**C**) Lactate levels (left panel) and mRNA levels of *Il4ra* and *Il10* (right panel) in livers of mice that left uninfected or i.p. infected with ~10^4^ CFU *Salmonella* WT or Δ*steE* for 3 days. (**D**) Flow cytometry analysis of M2 macrophage (CD68+CD163+) percentage from the liver of mice that were left uninfected or i.p. infected with ~10^4^ CFU *Salmonella* WT or Δ*steE* for 3 days. Representative dot plot and quantification of CD68+CD163+ macrophages are shown in the left and right panel, respectively. *Salmonella* colonization level from the same organs (solid black line, right y-axis) are shown. Data are presented as mean ± SD of three independent experiments (A–D). *P* values were determined using two-tailed unpaired Student’s *t*-test (**A, D**) or one-way ANOVA (**B, C**). ^*^ *P* < 0.05, ^**^ *P* < 0.01, ^***^ *P* < 0.001. ns, not significant.

We investigated the relationship between lactate- and *Salmonella*-mediated M2 macrophage polarization. *Salmonella* promotes macrophage M2 polarization via the SPI-2 T3SS effector SteE, which activates the STAT3 signaling pathway to stimulate the production of the critical anti-inflammatory cytokine IL10 (35). Interestingly, our previous study showed that macrophage-derived lactate could activate *Salmonella* SPI-2 gene expression, including *steE* (31). Consistent with the result, using a *steE*-lux transcriptional fusion and bioluminescent reporter assays, we showed that the addition of lactate induced the expression of *steE* in RAW264.7 cells (Fig. S2). Thus, we speculated that SteE is probably involved in the lactate-mediated macrophage M2 polarization during *Salmonella* infection. Macrophage infection assays showed that lactate addition increased *Salmonella* WT replication in RAW264.7 but did not influence the intracellular replication of Δ*steE* (Fig. 6A)*,* indicating that the mutation of *steE* abolished bacterial growth promotion mediated by lactate. Thus, *Salmonella* intracellular replication by lactate is SteE dependent. Quantitative real-time PCR (qRT-PCR) assays showed that lactate addition increased the expression of M2 macrophage marker genes (*Il4ra* and *Il10*) in *Salmonella* WT-infected RAW264.7 but did not influence the expression of M2 macrophage marker genes in Δ*steE*-infected RAW264.7 macrophages (Fig. 6B), indicating that the mutation of *steE* abolished the induction of macrophage M2 polarization by lactate. Thus, polarization of macrophages by lactate is SteE dependent.

Moreover, we analyzed the lactate levels and M2 marker gene expression in *Salmonella* WT- and Δ*steE*-infected mouse livers at day 3 post-infection. The results showed that lactate levels had increased in the liver of both WT-infected mice and Δ*steE*-infected mice compared to that of the uninfected mice (Fig. 6C, left panel), but relative expression of M2 marker genes was only increased in the livers of WT-infected mice but not in the livers of Δ*steE*-infected mice (Fig. 6C, right panel). Consistent with the expression of M2 marker genes, the percentage of M2 macrophages (CD163+, CD163 is commonly associated with M2 polarization) in the liver of WT-infected mice was significantly higher than that of Δ*steE*-infected mice (Fig. 6D). These results further indicate that SteE is required to promote M2 gene expression by lactate. The percentage of the CD163+ macrophage population correlated well with the bacterial burden of WT and Δ*steE* in the livers of infected mice (Fig. 6D), implying that SteE-mediated macrophage M2 polarization is required for *Salmonella* efficient colonization of mouse systemic tissues. Collectively, these results suggest that lactate promotes macrophage M2 polarization via SteE during *Salmonella* infection.

The relationship among lactate and SPI-2 expression, SteE translocation, and phosphorylation of STAT3 during *Salmonella* infection of macrophages was further investigated. Bioluminescent reporter assays revealed that lactate induced the higher expression of the *Salmonella* SPI-2 regulon (*ssaG*-lux) in RAW264.7 cells at 8 and 20 h post-infection, especially at 20 h post-infection (Fig. S3A). The addition of lactate increased the translocation of *steE*-FLAG to macrophage cytosol (Fig. S3B), as revealed by immunofluorescence analysis. Moreover, the translocation of SteE was quantified by Western blotting, using GAPDH as a loading control. The results showed that lactate increased the protein levels of SteE-FLAG in RAW264.7 cells (Fig. S3C and S4), further suggesting that lactate increased SteE translocation to RAW264.7 cells. In line with the increased SPI-2 expression and SteE translocation, Western blotting analysis showed that lactate increased the phosphorylation of STAT3 of RAW264.7 cells (Fig. S3D and S4). These results reveal that lactate drives higher expression of SPI-2 regulon, increased translocation of SteE, and, therefore, increased phosphorylation of STAT3, which in turn drives macrophage M2 polarization and promotes *Salmonella* intracellular replication.

## DISCUSSION

Replication within macrophages represents a critical step during the induction of systemic infection by *Salmonella* and requires complex crosstalk and interactions between *Salmonella* and host macrophages (36). More recent studies suggest that interactions with macrophages at the metabolic interface are important for *Salmonella* pathogenicity (37 – 40). In this study, we demonstrated that lactate promotes *Salmonella* intracellular replication and systemic infection by driving macrophage M2 polarization. The lactate-mediated macrophage M2 polarization depends on the function of the T3SS effector of *Salmonella*, SteE, which has been shown to activate the STAT3 signaling pathway (35, 41). Therefore, our results illustrate the mechanisms by which host-derived lactate promotes *Salmonella* pathogenicity and provides an additional perspective on host-pathogen crosstalk at the metabolic interface.

**Fig 7:**
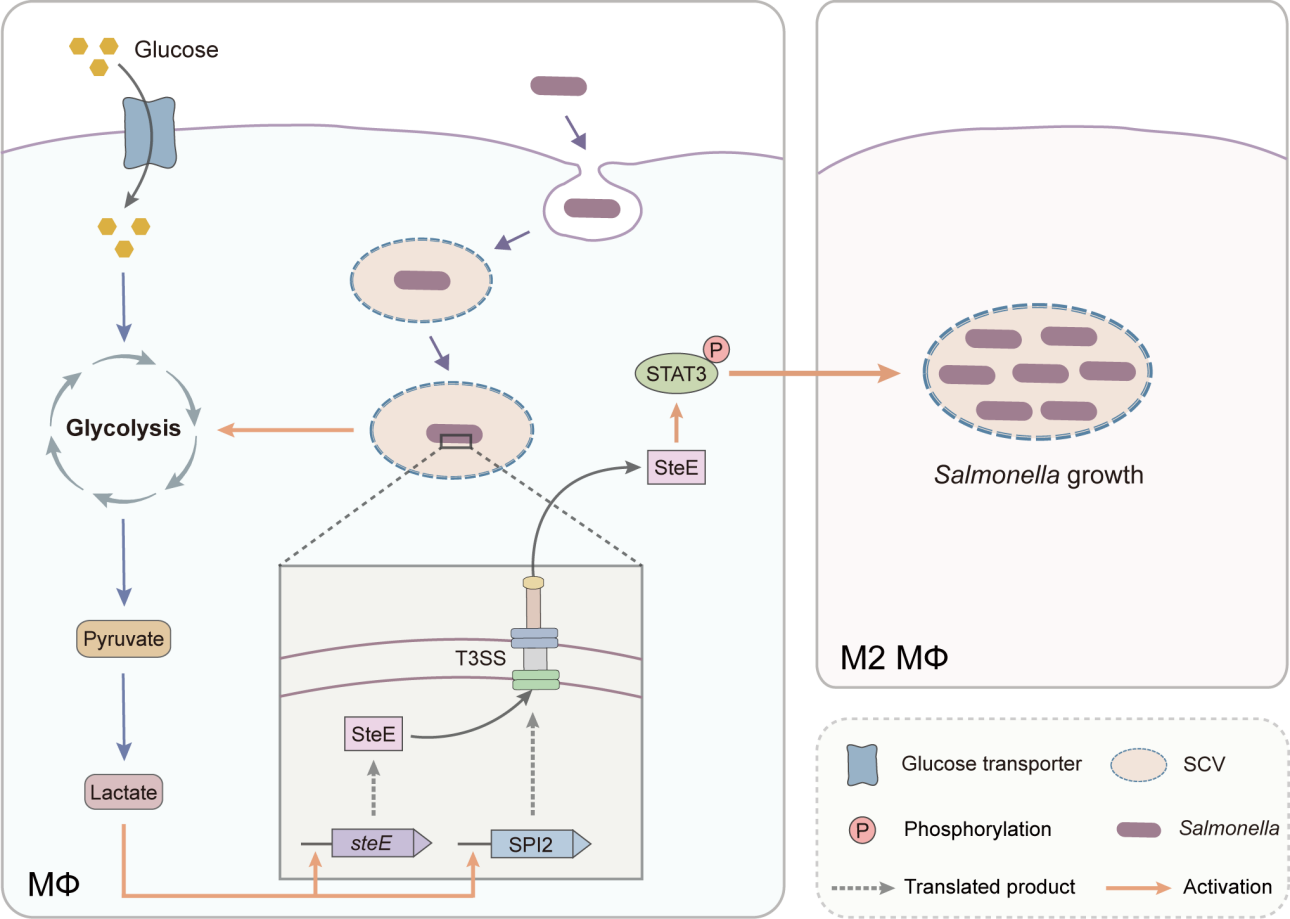
Model of lactate-mediated *Salmonella* growth in macrophages. *Salmonella* infection promotes macrophage glycolysis, and lactate levels increased in the infected macrophages. Host-derived lactate induces *Salmonella* SPI-2 T3SS expression, and the translocation of effector SteE from SCV to macrophage cytoplasm increased. SteE induces the phosphorylation of STAT3. Activated STAT3 drives macrophage M2 polarization, and *Salmonella* replicates in M2 macrophages. MФ, macrophage.

We propose a model for lactate-mediated *Salmonella* growth in macrophages (Fig. 7). After *Salmonella* enters the macrophages, macrophages reprogram their glucose metabolism, leading to an increase in glycolytic metabolism and lactate levels of the infected macrophages (42). Host-derived lactate is employed by intracellular *Salmonella* as a cue to promote SPI-2 T3SS expression (31), which leads to the increased translocation of effector SteE from SCV to macrophage cytoplasm. SteE induces the phosphorylation of STAT3, and then, the phosphorylated, activated STAT3 drives macrophage M2 polarization (24). The replication of *Salmonella* in M2 macrophages leads to bacterial dissemination and systemic infection. This model builds a direct link between lactate-mediated and *Salmonella*-directed macrophage M2 polarization and further highlights the complex interactions between *Salmonella* and macrophages.

Our findings suggest that lactate inhibits M1 bactericidal responses and increases M2 polarization of *Salmonella*-infected macrophages, promoting *Salmonella* replication in infected macrophages. However, lactate also accumulates in the extracellular environment during *Salmonella* infection, and its role in the surrounding macrophages, especially uninfected macrophages, remains unclear. Notably, during *in vivo* infection, a large proportion of macrophages are not infected with *Salmonella* (43). A previous study showed that lactate produced by tumor cells can promote the M2 polarization of tumor-associated surrounding macrophages to attenuate the engulfment of tumor cells by macrophages, contributing to tumor growth (44). Presumedly, *Salmonella* and other intracellular pathogens employ similar mechanisms to modulate M2 polarization of uninfected macrophages. Further investigations are necessary to determine the effects of lactate derived from *Salmonella*-infected macrophages on the polarized phenotype and immune function of the surrounding uninfected macrophages.

We showed that lactate-mediated macrophage M2 polarization depended on the SPI-2 T3SS effector SteE. Mutation of SteE abolished the induction of macrophage M2 polarization by lactate and *Salmonella* growth promotion mediated by lactate. SPI-2 loci are present in *Salmonella enterica* but absent in the genome of *Salmonella bongori*, which is predominantly associated with cold-blooded animals, and other intestinal bacterial pathogens (45). In addition, SteE is present primarily in *S*. Typhimurium but absent in many other *S. enterica* serovars, such as *S*. Enteritidis, *S*. Typhi and *S*. Paratyphi. We confirmed that the addition of lactate did not influence the replication of *S*. Enteritidis and *S*. Typhi in RAW264.7 macrophages (Fig. S5A and B). Thus, both lactate- and SteE-participating macrophage M2 polarization and the lactate-dependent increase in *Salmonella* replication are specific for *S*. Typhimurium but not for other *Salmonella* serovars, which lack the SteE effector. The mechanisms by which lactate promotes macrophage M2 polarization during infection with other bacterial pathogens require further study.

In addition to SteE, lactate also induces the expression of other SPI-2 effectors (31), for example, SifA, SseJ, SopD2, SseF, SseG, and SrfH, which are important for stimulating *Salmonella* replication in macrophages (46). However, it is unclear why the effect of lactate is specifically mediated via SteE but not mediated via other effectors. The different functions of these SPI-2 effectors lead to a plausible explanation that all these effectors are essential, while SteE acts in the “last step” for promoting *Salmonella* intracellular replication. First, SrfH inhibits the directed migration of infected macrophages to avoid their clearance by the host immune system (47). Second, SifA, SseJ, SopD2, SseF, and SseG induce the biogenesis and maintenance of the *Salmonella* replicative niche-SCV and/or the formation of *Salmonella*-induced filaments (SIFs) in infected macrophages (48 – 51), which provide the requirements supporting *Salmonella* persistence. Last, SteE drives macrophage M2 polarization via the phosphorylation of STAT3 (24), decreasing host inflammation and increased *Salmonella* replication. In addition, our results revealed a dose-dependent activity of SteE, with higher levels of SteE driving more STAT3 phosphorylation and more *Salmonella* replication. In contrast, the inhibition of macrophage migration and the formation of SCV (and SIFs) probably do not occur in dose-dependent manners. Therefore, although lactate induces the expression of many critical SPI-2 effectors, we only observed that its effect is mediated explicitly via SteE. Further research is needed to validate this hypothesis and illustrate the complex actions among SPI-2 effectors.

Lactate is one of the most abundant metabolites in host cells, and its abundance increases in infected macrophages due to the metabolic shift to a Warburg-like metabolism (52). In contrast to the induction of antibacterial effects upon metabolic reprogramming, M2 polarization induced by lactate promotes the anti-inflammatory functions of macrophages (53). Therefore, on the host side, lactate may balance the macrophage’s pro- and anti-inflammatory responses during bacterial infection to maintain tissue homeostasis. However, based on this study, we speculate that lactate-mediated homeostasis is exploited by *Salmonella*, which participate in the lactate-mediated M2 polarization pathway via the T3SS effector SteE.

Taken together, our results suggest that host-derived lactate promotes *Salmonella* intracellular replication and systemic infection by driving macrophage M2 polarization. In addition to the host STAT3 transcription factors, the *Salmonella* T3SS effector SteE is also required for lactate-directed M2 polarization, indicating that *Salmonella* actively regulates host cell polarization to expand the population of permissive macrophages for their survival. In addition to *Salmonella,* many other intracellular bacteria use macrophages for replication; links to macrophage M2 polarization and other bacterial infection require further investigations.

## MATERIALS AND METHODS

### Mice

Female C57BL/6 mice (6–8 weeks old) were obtained from Beijing Vital River Laboratory Animal Technology Co., Ltd. (Beijing, China). *LdhA^flox/flox^* and *Albumin-Cre* mice (C57BL/6 genetic background) were obtained from Gempharmatech Co., Ltd. (Jiangsu, China). Liver-specific LdhA knockout mice (LDHA^−/−^) were generated via crossing *LdhA^flox/flox^* mice with *Albumin-Cre* mice. Mice were maintained under specific pathogen-free conditions, with a 12-h light–dark cycle (temperature: 24°C ± 2°C; relative humidity: 50% ± 5%). All experimental procedures were approved by the Institutional Animal Care Committee of Nankai University (2021-SYDWLL-000029), Tianjin, China.

### Bacterial strains, plasmids, and growth conditions

The bacterial strains and plasmids used in this study are listed in Table S1. *Salmonella enterica* serovar Typhimurium ATCC 14028s was used as the wild-type strain throughout this work. Mutant strains and FLAG-tagged strain (*steE*-FLAG) were generated using the λ Red-based recombination system (54, 55). To generate the *steE*-lux transcriptional fusion, the PCR products of *steE* promoter region were digested with BamHI and XhoI and cloned into the XhoI–BamHI site of the plasmid pMS402, which carries a promoter-less *luxCDABE* reporter gene cluster. The *steE*-lux fusion plasmid was then transformed into WT strain to generate the *lux* reporter strain WT+*steE-*lux. The primers used to construct the strains and plasmids are listed in Table S2. *Salmonella* strains were grown to stationary phase in Luria-Bertani (LB) broth at 37°C with aeration for infection of macrophages and mouse infection assays. When required, antibiotics were added at the following final concentrations: 25 µg/mL chloramphenicol, 100 µg/mL ampicillin, and 50 µg/mL kanamycin.

### Cell culture

The mouse macrophage cell line RAW264.7 (ATCC TIB71) was obtained from the Cell Bank of the Chinese Academy of Sciences (Shanghai, China) and cultured in Dulbecco's Modified Eagle Medium (DMEM) supplemented with 10% fetal bovine serum (FBS) at 37°C with 5% CO_2_. BMDMs were prepared by flushing the femurs and tibia of C57BL/6 wild-type mice with PBS. Harvested BMDMs were differentiated in DMEM containing 10% FBS and 10  ng/mL Macrophage Colony Stimulating Factor (M-CSF) (Proteintech, HZ-1192). Cells were incubated for 7 days at 37°C in a 5% CO_2_ environment with medium change every 2–3 days.

### Macrophage infections

BMDMs or RAW264.7 cells were seeded at a density of 1 × 10^5^ cells per well in a 24-well culture plate and infected with *Salmonella* WT or mutant strains grown to stationary phase at a multiplicity of infection of 10. When indicated, the cells were treated with 10 ng/mL recombinant mouse IL4 (Peprotech, 214–14) for 24 h before infection. Overnight-cultured bacterial strains were serially diluted to 1 × 10^6^ colony-forming units (CFUs)/mL, opsonized in DMEM containing 10% serum for 30 min, and then added to macrophage monolayers. The plates were centrifuged at 500 × *g* for 5 min to synchronize bacterial uptake. After incubation for 30 min, the cells were washed thrice with PBS and incubated in DMEM containing 100 µg/mL gentamicin for 1 h to remove extracellular bacteria. The cells were then incubated in a DMEM containing 10 µg/mL gentamicin (Gm) for the remainder of the experiment. Infected cells were lysed using 1% Triton X-100 at 2 and 20 h post-infection, and intracellular bacteria were enumerated on LB plates for CFU analysis. Fold intracellular replication of *Salmonella* strains was determined by dividing the intracellular bacterial load recovered at 20 h by that recovered at 2 h. A 3 mM lactate and/or a 23.3 µM FX11 were added after 1 h of gentamicin treatment to avoid the possible influence of lactate (and/or FX11) on bacterial internalization.

### Immunofluorescence microscopy

To enumerate intracellular bacteria, BMDMs seeded on 20-mm-diameter coverslips were infected with *Salmonella* WT as described above. A 3 mM lactate was added after 1 h of gentamicin treatment. At 2 and 20 h post-infection, the cells were washed thrice with PBS, fixed with 4% paraformaldehyde (PFA; Solarbio, P1110) at 4°C for 15 min and blocked with 5% bovine serum albumin (BSA; Solarbio, SW3015) at room temperature for 1 h. The cells were then incubated with the primary antibody mouse anti-*Salmonella* Typhimurium LPS (Abcam, ab8274; 1:100 dilution) at room temperature for 1 h. Next, the cells were washed thrice with PBS and incubated with fluorophore-conjugated secondary antibody Goat Anti-Mouse IgG H&L (Alexa Fluor 488) (Abcam, ab150113; 1:200 dilution) at room temperature for 1 h. After being washed thrice with PBS, the cell nuclei were stained with 5% DAPI (Bioss, C02-04002) at room temperature for 5 min. For detecting the translocation of SteE, RAW264.7 cells seeded on 20-mm-diameter coverslips were infected with WT *steE*-FLAG strain for 8 and 20 h. The cells were then incubated with the primary antibodies FITC Anti-*Salmonella* antibody (Abcam, ab20320; 1:50 dilution), Recombinant Alexa Fluor 647 Anti-LAMP1 antibody (Abcam, ab237307; 1:100 dilution), and Recombinant Anti-DDDDK tag (Binds to FLAG tag sequence) antibody (Abcam, ab205606; 1:100 dilution) at room temperature for 1 h. Next, the cells were washed thrice with PBS and incubated with fluorophore-conjugated secondary antibody Goat Anti-Rabbit IgG H&L (Alexa Fluor 405) (Abcam, ab175652; 1:500 dilution) at room temperature for 1 h. The stained cells were visualized using a Zeiss LSM800 confocal microscope (Zeiss, Germany). The ZEN 2.3 (blue edition) was used for the further image processing.

### Mouse infections

To enumerate bacterial burden in mouse liver and spleen, mice were intraperitoneally (i.p.) infected with ~10^4^ CFUs of the WT strain plus lactate (0.6 mg lactate/g mice) or bacteria only in PBS. The infected mice were sacrificed on day 3 post-infection. Their livers and spleens were collected, homogenized in PBS, serially diluted, and plated on LB agar plates to obtain CFUs per gram of tissue. To detect M2 marker gene expression in mouse livers, the infected mice were sacrificed on day 3 post-infection. The livers were homogenized with TRIzol reagent to extract total RNA for subsequent qRT-PCR experiments.

### Measurement of lactate concentration

To assess the lactate levels of BMDMs, cells were left uninfected or infected with *Salmonella* WT for 20 h, and the supernatants were collected to measure lactate concentration. To assess lactate levels in the mouse liver, mice were left uninfected or intraperitoneally infected with ~10^4^ CFU *Salmonella* WT or *steE* mutant. The infected mice were sacrificed on day 3 post-infection, and their spleens were collected and homogenized in 1 mL of PBS to measure lactate concentration. The lactate concentration was measured using a lactate assay kit (Sigma-Aldrich, MAK064) according to the manufacturer’s instructions.

### Seahorse analysis

BMDMs were seeded into Seahorse 24-well tissue culture plates (Agilent Technologies) and left uninfected or infected with *Salmonella* WT for 20 h in the presence or absence of 3 mM lactate. The OCR and ECAR of BMDMs were measured on the Seahorse XF24 Analyzer (Agilent Technologies), by using Seahorse XF Glycolysis Stress Test Kit (Agilent Technologies, 103020) and Seahorse XF Cell Mito Stress Test Kit (Agilent Technologies, 103015), respectively, according to manufacturer’s instructions. The basal OCR was calculated by subtracting non-mitochondrial respiration from the values obtained before oligomycin addition. The ECAR was calculated by subtracting the normalized ECAR values after 2-deoxy-D-glucose injection from the ECAR values after glucose injection.

### RNA isolation and quantitative real-time PCR

Total RNA was isolated using the RNAsimple Total RNA Kit (TIANGEN Biotech, DP419), and cDNA was synthesized using StarScript III RT Master Mix (Genstar, A233) according to the manufacturer’s instructions. Each 20-µL qRT-PCR reaction system contained 1-µL cDNA, 10 µM of each primer, and 10 µL of 2× RealStar Power SYBR qPCR Mix (Genstar, A314). The qRT-PCR was run on a QuantStudio Real-Time PCR Systems (Thermo Fisher Scientific) using the following program: 95°C for 10 min, followed by 40 cycles of 95°C for 15 s and 60°C for 30 s. Expression of target genes was normalized to *Gapdh* as a control. Relative changes in gene expression were analyzed using the comparative cycle threshold (ΔΔCt) method (56).

### Small interfering RNA (siRNA) transduction

*ldhA* and control siRNAs were designed and synthesized by Guangzhou RiboBio Co., Ltd. (GuangZhou, China). Primer for siRNA against *ldhA*:5′–GGAATCAATGAGGATGTCT–3′. RAW264.7 cells were transfected with *ldhA* siRNA or control siRNA at a final concentration of 50 nM using RiboFECT CP Transfection Kit (RiboBio, C10511) 48 h before bacterial infection. The interference efficiency was assessed via qRT-PCR at 24 h after transfection.

### Bioluminescent reporter assays

To determine the effect of lactate on intracellular *ssaG* and *steE* expression, RAW264.7 cells were infected with the WT+*ssaG-*lux or WT+*steE-*lux. A 3-mM lactate was added after 1 h of gentamicin treatment. At 8 and 20 h post-infection, the cells were lysed with 1% Triton X-100. A 200-µL sample of the cell lysates was loaded into a 96-well black assay plate with a clear flat bottom (Corning 3603) to measure luminescence using the Spark multimode microplate reader (Tecan). A 100-µL sample of the cell lysates was serially diluted and plated on LB agar plates to enumerate intracellular bacterial CFUs. Luminescence values were normalized to intracellular bacterial CFUs.

### Flow cytometry

Mice were i.p. infected with ~10^4^ CFUs of *Salmonella* WT or Δ*steE*, with two mice in each bacterial injection group. The WT- and Δ*steE*-infected mice were sacrificed on day 3 post-infection. Their livers were collected and divided into two parts with scissors on each liver, one for detecting bacterial CFUs and the other for flow cytometry analysis. For flow cytometry analysis, liver from two mice (of one group) were pooled together and then dissociated into single cells through gentleMACS/Mouse liver dissociation kit (Miltenyi Biotec, 130-105-807). Cells were fixed with 4% PFA and permeabilized with 0.1% Triton X-100. The fixed cells were then stained with an anti-CD68 antibody (Abcam, ab221251), an anti-CD163 antibody (Abcam, ab182422), and goat anti-rabbit lgG H&L (FITC) (Abcam, ab6717). The cells were suspended in PBS and adjusted to a concentration of 1 × 10^5^  cells/mL. Nonspecific antigens were blocked using a 5% BSA buffer. The cells were analyzed using a BD FACSAria Flow Cytometer (BD Biosciences). To determine bacterial CFUs, livers from two mice (of one group) were pooled and homogenized in PBS together, serially diluted, and plated on LB agar plates to obtain CFUs per gram. Three independent experiments were performed.

To prepare whole cell lysates, cells were washed once in PBS and lysed in SDS loading buffer before sonication. For post nuclear supernatant (PNS) and pellet analysis, cells were washed once in PBS and lysed in cell lysis buffer (10% glycerol, 20 mM Tris-Cl pH 7.4, 150 mM NaCl, 0.1% Triton X-100) on ice for 10 min before clarification by centrifugation. SDS loading buffer was added to the PNS and pellet samples so that both fractions were in the same final volume. All samples were then heated to 95°C and separated by SDS-PAGE using either 8%, 10%, or 12% polyacrylamide denaturing gels, transferred to polyvinylidene difluoride (PVDF) membrane (Millipore), and visualized by immunoblotting using ECL detection reagents (Dako) on a Chemidoc Touch Imaging System (Bio-Rad).

### Western blotting

RAW264.7 cells were infected with WT strain. A 3 mM lactate was added after 1 h of gentamicin treatment. At 8 and 20 h post-infection, the infected cells were lysed in radioimmunoprecipitation assay buffer (Sigma #R0278) on ice for 10 min and then centrifuged at 12,000 rpm for 10 min at 4°C. The supernatants were collected, mixed with SDS loading buffer, boiled for 10 min, and then separated by a SurePAGE gel (GenScript #M00658). Separated proteins were transferred to PVDF membranes and blocked with TBST [Tris-buffered saline with 0.1% (vol/vol) Tween 20] containing 5% skim milk for 2 h at room temperature. Membranes were incubated with the primary antibodies [Stat3 (D3Z2G) Rabbit mAb, 1:1,000 dilution, Cell Signaling Technology #12640; Phospho-Stat3 (Tyr705) (D3A7) XP Rabbit mAb, 1:2,000 dilution, Cell Signaling Technology #9145; GAPDH (D16H11) XP Rabbit mAb, 1:1,000 dilution, Cell Signaling Technology #5174], followed by the secondary antibody Goat Anti-Rabbit IgG(H + L) HRP (1:5,000 dilution, Sparkjade #EF0002). Immunoreactions were detected using BeyoECL Star Ultra-sensitive ECL chemiluminescence kit (Beyotime #P0018AS). Images were acquired using an Amersham Imager 600 System (General Electric Company). Western blotting bands were quantified using Image J180 software.

### Data analysis

Data were obtained from three independent experiments and were presented as mean ± SD, unless otherwise indicated. Statistical significance was determined by two-tailed unpaired Student’s *t*-test, Mann-Whitney *U* test, one-way analysis of variance (ANOVA), or two-way ANOVA using GraphPad Prism 8.0.1 software (GraphPad, Inc., San Diego, CA, USA). Significant differences between groups are represented as ^*^ *P* < 0.05, ^**^ *P* < 0.01, and ^***^ *P* < 0.001. ns represents no statistical significance.

## Data Availability

The data that support the findings of this study are provided in Supplemental File 2.

## SUPPLEMENTAL MATERIAL

Supplemental figures and tables spectrum.02253-23-s0001.pdf Fig. S1 to S5; Tables S1 and S2.

Supplemental dataset spectrum.02253-23-s0002.xlsx Supporting data for Fig. 1 to 6 and Fig. S1 to S5.

ASM does not own the copyrights to Supplemental Material that may be linked to, or accessed through, an article. The authors have granted ASM a non-exclusive, world-wide license to publish the Supplemental Material files. Please contact the corresponding author directly for reuse.
