# Supplementary material for: Lactate promotes Salmonella intracellular replication and systemic infection via driving macrophage M2 polarization
Source: Microbiol Spectr. 2023 Oct 5;11(6):e02253-23. doi: 10.1128/spectrum.02253-23 (PMC10715217; doi:10.1128/spectrum.02253-23)
Supplement: Supplemental figures and tables — Fig. S1 to S5; Tables S1 and S2. [file spectrum.02253-23-s0001.pdf]

SUPPLEMENTAL MATERIAL

Lactate Promotes *Salmonella* Intracellular Replication and Systemic Infection via Driving Macrophage M2 Polarization

Xinyue Wang[a,†], Bin Yang[a,†], Shuangshuang Ma[a,b,†], Xiaolin Yan[a], Shuai Ma[a], Hongmin Sun[a], Yuyang Sun[a], Lingyan Jiang[a,*]

[a]*The Key Laboratory of Molecular Microbiology and Technology, Ministry of Education, TEDA Institute of Biological Sciences and Biotechnology, Nankai University, Tianjin 300457, China;* [b]*Tianjin Key Laboratory on Technologies Enabling Development of Clinical Therapeutics and Diagnostics, Department of Biopharmaceuticals, School of Pharmacy, Tianjin Medical University, Tianjin 300070, China.*

[†] Xinyue Wang, Bin Yang and Shuangshuang Ma contributed equally to this work.

[*]Correspondence and requests for materials should be addressed to Lingyan Jiang (email: jianglingyan@nankai.edu.cn).

Table of contents

Fig S1, S2, S3, S4, S5

Table S1, S2

FIG S1

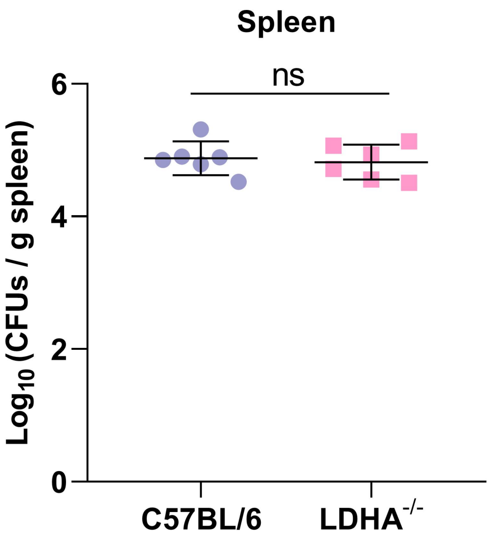

FIG S1 Bacterial counts recovered from the spleen of wild-type or liver-specific LDHA knockout (LDHA$^{-/-}$) C57BL/6 mice i.p. infected with *Salmonella* WT. Mice were i.p injected with ~10^4 CFU bacteria. The spleen was collected on day 3 post-infection to quantify bacterial burden. n = 6 mice per group. *p* values were determined using Mann–Whitney U test. ns, not significant.

FIG S2

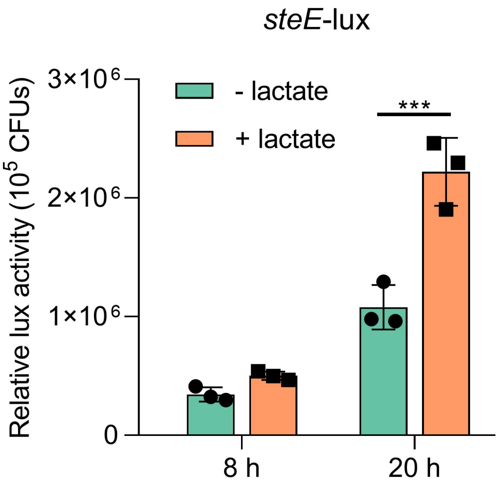

FIG S2 Lactate induces the expression of *steE*. RAW264.7 cells were infected with the WT+*steE*-lux strain at a MOI of 10, in the presence or absence of 3 mM lactate. The cells were lysed at 8 and 20 h post-infection, and luminescence and CFU counts were determined. Luminescence values were normalized to intracellular bacterial CFUs. Data are presented as mean ± SD of three independent experiments. *p* values were determined using two-way ANOVA. $^{***}p < 0.001$.

FIG S3

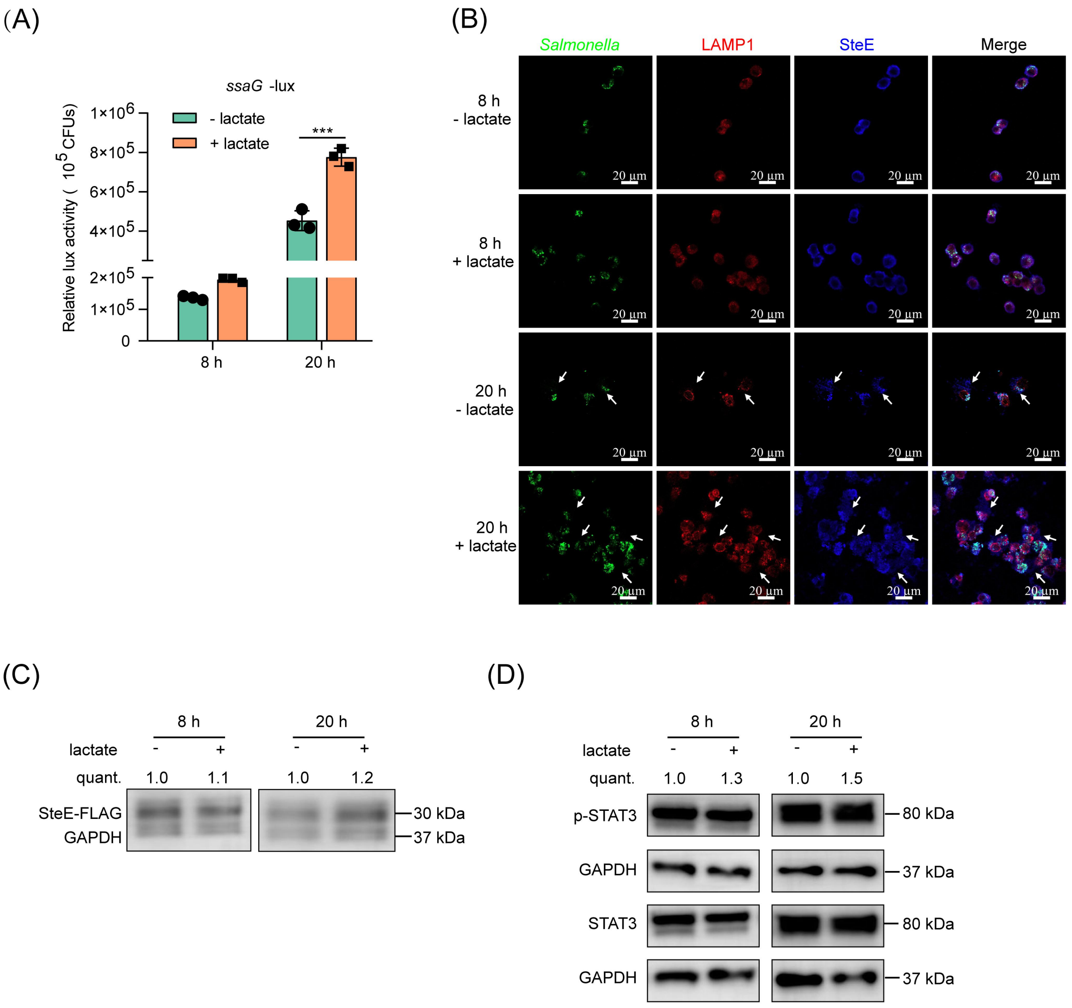

FIG S3 Lactate promotes SPI-2 expression, SteE translocation, and the phosphorylation of STAT3 during *Salmonella* infection of macrophages. (A) Expression of the *ssaG*-lux transcriptional fusion in macrophages. RAW264.7 cells were infected with the WT+*ssaG*-lux strain, in the presence or absence of 3 mM lactate. The cells were lysed at 8 and 20 h post-infection and luminescence and CFU counts were determined. Luminescence values were normalized to intracellular bacterial CFUs. Data are presented as mean ± SD of three independent experiments. *p* values were determined using two-way ANOVA. $^{***}p < 0.001$. (B)

Immunofluorescence analysis of the translocation of SteE. RAW264.7 cells seeded on 20 mm-diameter coverslips were infected with *Salmonella steE*-FLAG strain for 8 and 20 h. Samples were stained for *Salmonella* using a FITC Anti-*Salmonella* antibody (green), for LAMP-1 using an Alexa Fluor® 647 Anti-LAMP1 antibody (red), and for the FLAG-tagged SteE using an Anti-FLAG antibody and an Alexa Fluor® 405-conjugated secondary antibody (blue). The localization of *Salmonella*, LAMP-1, and SteE is represented by green, red, and blue colors, respectively. Scale bars, 20 μm. Images are representative of three independent experiments. (C) Western blotting quantify the translocation of SteE. RAW264.7 cells were infected with the *Salmonella steE*-FLAG strain, in the presence or absence of 3 mM lactate. At 8 and 20 h post-infection, the cells were collected, lysed, and used for western blotting. The number above each band denotes the band intensity after normalization with GAPDH. (D) Western blotting analysis the phosphorylation of STAT3. RAW264.7 cells were infected with the WT+*ssaG*-lux strain, in the presence or absence of 3 mM lactate. At 8 and 20 h post-infection, the cells were lysed and used for western blotting. The number above each band denotes the band intensity after normalization with GAPDH. Images are representative of three independent experiments. Full gels of the western blotting are shown in Fig S4 (C, D).

FIG S4

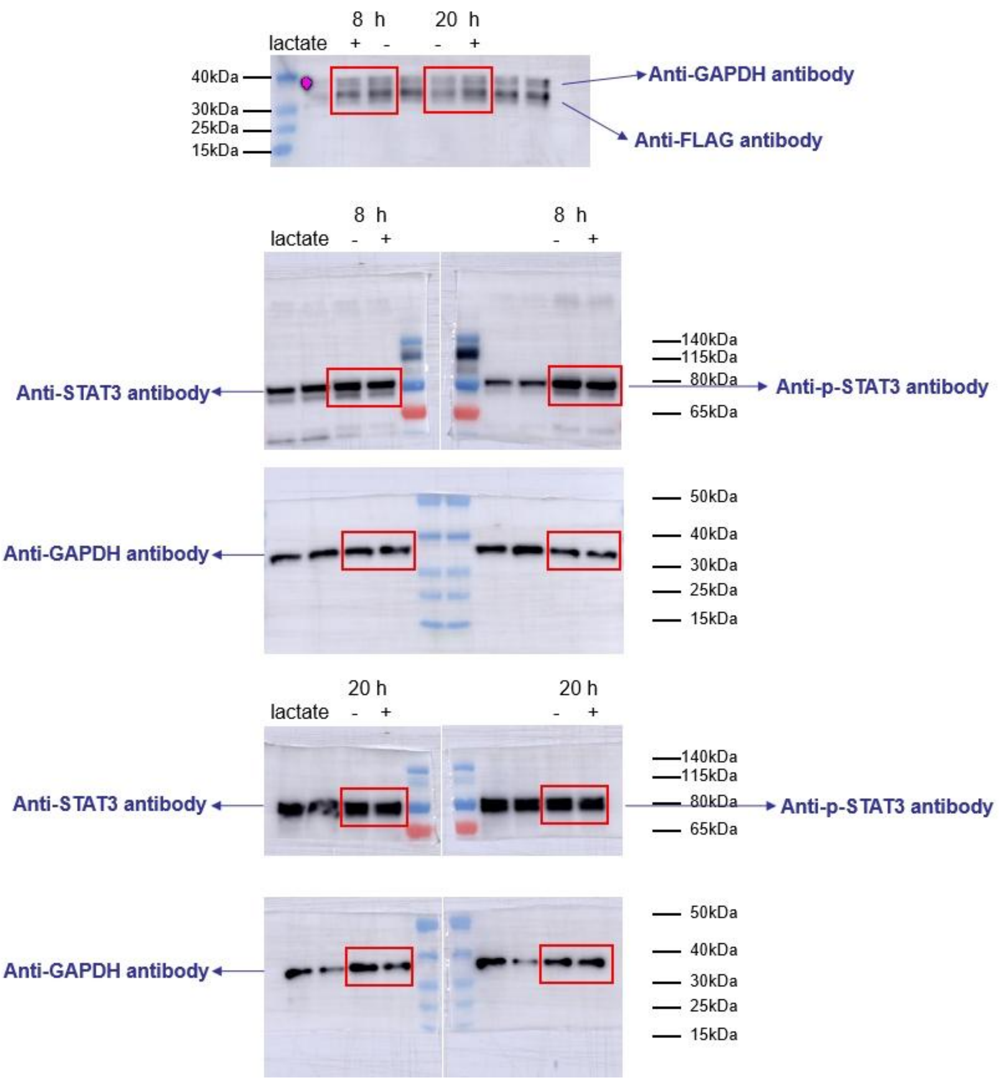

FIG S4 Full gels of the western blotting. Boxes highlight lanes used in the Fig. S3C and S3D.

FIG S5

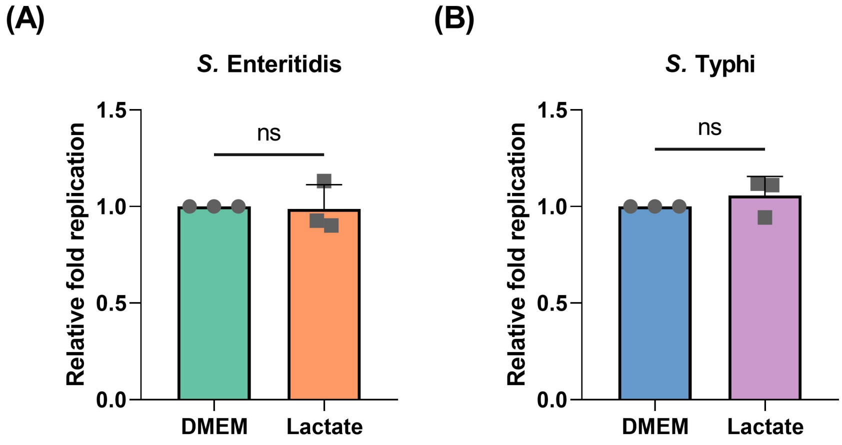

FIG S5 Lactate does not influence the replication of *S*. Enteritidis and *S*. Typhi in macrophages. Replication of *S*. Enteritidis (A) and *S*. Typhi (B) in RAW264.7 cells, in the presence or absence of 3 mM lactate. Data are presented as mean ± SD of three independent experiments (A, B). *p* values were determined using two-tailed unpaired Student's *t*-test (A, B). ns, not significant.

Table S1 Strains and plasmids used in this study.

Strains	Genotype or description	Source
WT *S.* Tm	Wild-type *S.* Typhimurium ATCC 14028s	ATCC
S. Enteritidis	Wild-type *Salmonella* enterica serovar Enteritidis CMCC50041	CMCC
S. Typhi	Wild-type *Salmonella* enterica serovar Typhi Ty2	ATCC
Δ*lldD*	WT *S.* Tm strain *lldD*::Cm; Cm^R	This study
Δ*ldhA*	WT *S.* Tm strain *ldhA*::Cm; Cm^R	This study
Δ*dld*	WT *S.* Tm strain *dld*::Km; Km^R	This study
Δ*ldhA*Δ*dld*	WT *S.* Tm strain *ldhA*::Cm and *dld*::Km; Cm^R, Km^R	This study
Δ*lldD*Δ*ldhA*Δ*dld*	WT *S.* Tm strain *ldhA*, *dld* deletion and *lldD*::Cm; Cm^R	This study
Δ*steE*	WT *S.* Tm strain *steE*::Cm; Cm^R	This study
WT+*ssaG*-lux	WT *S.* Tm containing plasmid *ssaG*-lux; Km^R	Lab construction
WT+*steE*-lux	WT *S.* Tm containing plasmid *steE*-lux; Km^R	This study
steE-FLAG	WT *S.* Tm *steE* gene tagged with FLAG tag; Cm^R	This study
**Plasmids**		
pKD46	λ-Red recombinase system under an arabinose-inducible promoter; Ap^R	Lab collection
pKD3	template plasmid containing a chloramphenicol resistance cassette; Cm^R	Lab collection
pKD4	template plasmid containing a kanamycin resistance cassette; Km^R	Lab collection
pCP20	temperature-sensitive replicon expressing the FLP gene to abolish the antibiotic resistance of the mutant strains; Ap^R	Lab collection
pMS402	containing a promoterless *luxCDABE* reporter gene cluster; Km^R	Lab collection
ssaG-lux	pMS402 carrying the *ssaG* gene promoter region; Km^R	Lab construction
steE-lux	pMS402 carrying the *steE* gene promoter region; Km^R	This study

ATCC, American Type Culture Collection; CMCC, National Center for Medical Culture Collections (Beijing, China).

Table S2 Primers used in this study (5'–3')

**Primers for gene mutation**		
lldD	F	AGGCGCGTATTACCCGCCTGCCCGGCGACCATAATGAGATGACCAGGGAGAATAAATCGTGTAGGCTGGAGCTGCTTCG
	R	CCCCTTAAATGCGGGGGCAGAATAGCAGAAATTTACCGGGCTCTCACGCGCCAGGCGTCATATGAATATCCTCCTTAG
ldhA	F	TTTTGTAAGATAATTTTAAGCATATTTGTGTGATTCAAACATCACTGGAGAAGGTCATGTGTAGGCTGGAGCTGCTTCG
	R	CGGATAGATCTTCAGGGGTTATCCGAAAATGCTCCCCTGAGCGCAGGGGAGCGACAGGACATATGAATATCCTCCTTAG
dld	F	TGTCATACAAAGCGCTATGCTTAACGCTGATATTTTGTCCCACCACAAGGAGTGGAGAGTGTAGGCTGGAGCTGCTTCG
	R	AGTCGCCGCAGGCCCGGCAGGCATCGCGCCGCCGGGCTTCACGGTAAACCGCACGGTGCATATGAATATCCTCCTTAG
steE	F	CGGACTGAGTTCAATCAAAGTGATCTACTATTCGGCGCAGCTATTTATAACGCTTTGTTGTGTAGGCTGGAGCTGCTTCG
	R	AACAGGCTTGAATTACTTTTCTTTAAAAACTACTGCATGTAAAAGGGTCTCCTCTTGTTCATATGAATATCCTCCTTAG
steE-FLAG	F	CGCTCAATACAGGCATTCTGCAGAGGTTTTCCCGGATGAAGATTACAAGGATGACGACGATAAGTAACATATGAATATCCTCCTTAG
	R	ACAATCCGGACTGAGTTCAATCAAAGTGATCTACTATTCGGCGCAGCTATTTATAACGCTTTGTTGTGTAGGCTGGAGCTGCTTCG
**Primers for gene cloning**		
ssaG-lux	F	CCGCTCGAGAGCCTCATTTATTAGAGCGT
	R	CGGGATCCCTGGGTTGAGCAAATCATT
steE-lux	F	CCGCTCGAGAAGGGCAATCTGAGTATTTACAGG
	R	CGGGATCCCTCGCCACCCTGTTAGTACT

Primers for qRT-PCR		
Gapdh	F	TGTAGACCATGTAGTTGAGGTCA
	R	AGGTCGGTGTGAACGGATTTG
Il4ra	F	ACCAGATGGAACTGTGGGCTGA
	R	AGCAGCCATTCGTCGGACACAT
Il10	F	CGGGAAGACAATAACTGCACCC
	R	CGGTTAGCAGTATGTTGTCCAGC
Il1β	F	TGGACCTTCCAGGATGAGGACA
	R	GTTCATCTCGGAGCCTGTAGTG
Tnf	F	GGTGCCTATGTCTCAGCCTCTT
	R	GCCATAGAACTGATGAGAGGGAG
iNOS	F	CCTCTTTCAGGTCACTTTGGTAGG
	R	TTGGGTCTTGTTCAGCCACGG
ldhA	F	GATGGCAACCCTCAAGGACC
	R	TCATCCGCCAAGTCCTTCATT
